# The Hair-Tonic Bottle

**Published:** 1913-06

**Authors:** Ben King


### The Hair-Tonic Bottle.

How dear to my heart is the old village drug store,
  When tired and thirsty it comes to my view.
The wide-spreading sign that asks you to "Try it,"
  Vim, Vaseline, Vermifuge, Hop Bitters, too.
The old rusty stove and the spittoon by it,
  That little back room. Oh! you've been there yourself,
And ofttimes have gone for the doctor's prescription,
  But tackled the bottle that stood on the shelf.

The friendly old bottle,
The plain-labeled bottle,
The "Hair-Tonic" bottle that stood on the shelf.

How oft have I seized it with hand that were glowing,
  And guzzled awhile ere I set off for home;
I owned the earth all that night, but next morning
  My head felt as big as the Capitol's dome.
And then how I hurried away to receive it,
  The druggist would smile o'er his poisonous pelf,
And laugh as he poured out his unlicensed bitters,
  And filled up the bottle that stood on the shelf.

The unlicensed bottle,
The plain-labeled bottle,
That "Hair-Tonic" bottle that stood on the shelf.

—Ben King.

